# Sarcopenia in atrial fibrillation: a risk factor for adverse outcomes in a UK Biobank study

**DOI:** 10.1093/europace/euaf286

**Published:** 2025-11-08

**Authors:** Hong-Ju Kim, Pil-Sung Yang, Hanjin Park, Daehoon Kim, Han-Joon Bae, Chan-Hee Lee, Jang-won Son, Ung Kim, Boyoung Joung

**Affiliations:** Division of Cardiology, Department of Internal Medicine, Severance Cardiovascular Hospital, Yonsei University College of Medicine, 50-1 Yonsei-ro, Seodaemun-gu, Seoul 03722, Republic of Korea; Division of Cardiology, Department of Internal Medicine, Yeungnam University Medical Center, Yeungnam University College of Medicine, Daegu, Republic of Korea; Division of Cardiology, Department of Internal Medicine, Severance Cardiovascular Hospital, Yonsei University College of Medicine, 50-1 Yonsei-ro, Seodaemun-gu, Seoul 03722, Republic of Korea; Division of Cardiology, Department of Internal Medicine, Severance Cardiovascular Hospital, Yonsei University College of Medicine, 50-1 Yonsei-ro, Seodaemun-gu, Seoul 03722, Republic of Korea; Division of Cardiology, Department of Internal Medicine, Severance Cardiovascular Hospital, Yonsei University College of Medicine, 50-1 Yonsei-ro, Seodaemun-gu, Seoul 03722, Republic of Korea; Department of Cardiology, Daegu Catholic University School of Medicine, Daegu, Republic of Korea; Division of Cardiology, Department of Internal Medicine, Yeungnam University Medical Center, Yeungnam University College of Medicine, Daegu, Republic of Korea; Division of Cardiology, Department of Internal Medicine, Yeungnam University Medical Center, Yeungnam University College of Medicine, Daegu, Republic of Korea; Division of Cardiology, Department of Internal Medicine, Yeungnam University Medical Center, Yeungnam University College of Medicine, Daegu, Republic of Korea; Division of Cardiology, Department of Internal Medicine, Severance Cardiovascular Hospital, Yonsei University College of Medicine, 50-1 Yonsei-ro, Seodaemun-gu, Seoul 03722, Republic of Korea

**Keywords:** Sarcopenia, Atrial fibrillation, All-cause mortality, Major bleeding, Stroke, UK Biobank

## Abstract

**Aims:**

Sarcopenia, characterized by reduced muscle mass and function, has been increasingly implicated in cardiovascular disorders. However, its prognostic relevance in atrial fibrillation (AF) remains unclear. We aimed to evaluate the association between sarcopenia and adverse outcomes in individuals with AF using UK Biobank data.

**Methods and results:**

This retrospective cohort study included individuals with AF enrolled between 2006 and 2010 at 22 centres. Sarcopenia was defined per European Working Group on Sarcopenia in Older People 2 (EWGSOP2) criteria as low muscle strength and/or low muscle mass measured by handgrip and bioelectrical impedance analysis. Propensity score weighting adjusted for baseline differences. The primary outcome was a composite of all-cause mortality, major bleeding, thromboembolic events (stroke/systemic embolism), and heart failure admission; each component was also assessed individually. Among 5144 patients with AF (median age, 64.0 years; 24.1% female), 16.7% had sarcopenia. After propensity score weighting, sarcopenia was associated with a higher incidence of the primary composite outcome [43.9 per 1000 person-years (PYRs)], with an adjusted hazard ratio (HR) of 1.30 [95% confidence interval (CI), 1.15–1.46]. This risk was mainly driven by elevated rates of all-cause mortality (26.4 per 1000 PYRs; aHR, 1.44; 95% CI 1.24–1.68) and major bleeding (14.4 per 1000 PYRs; aHR, 1.34; 95% CI 1.10–1.65). Subgroup analyses demonstrated consistent results.

**Conclusion:**

Even after PS weighting analysis, some residual confounders may remain; however, sarcopenia was independently associated with adverse clinical outcomes, particularly mortality and bleeding risk. Screening for sarcopenia may enhance risk stratification and management, particularly in patients receiving anticoagulation.

## Graphical Abstract

**Graphical Abstract:**
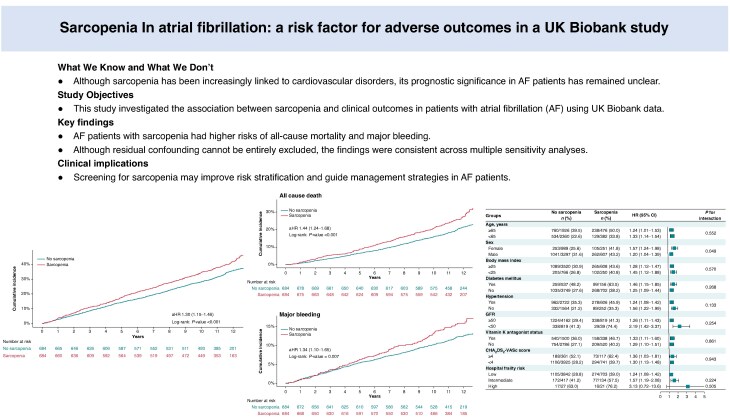

What’s new?

- This study investigated the association between sarcopenia and clinical outcomes in patients with atrial fibrillation (AF) using UK Biobank data.
- Although sarcopenia has been increasingly linked to cardiovascular disorders, its prognostic significance in AF patients has remained unclear.
- In the study cohort, AF patients with sarcopenia had higher risks of all-cause mortality and major bleeding.
- Although potential confounders may remain even after propensity score weighting analysis, the results were robust across multiple sensitivity analyses.
- The findings suggest that screening for sarcopenia may improve risk stratification and guide management strategies in AF patients.

## Introduction

Atrial fibrillation (AF) is increasingly prevalent in aging populations and is anticipated to affect up to 12.1 million individuals in the USA and 5.4% of the population in Asia by 2050.^1,2^ The management of AF has evolved from the traditional ABC pathway to the AF-CARE model, as outlined in contemporary clinical guidelines.^3–6^ Current therapeutic strategies emphasize anticoagulation to prevent thromboembolism, rate or rhythm control for symptom relief, and addressing comorbid conditions. Notably, recent guidelines highlight the importance of identifying and modifying risk factors for bleeding in anticoagulated patients with AF. These recommendations emphasize a multidisciplinary patient-oriented approach tailored to specific populations, such as individuals with sarcopenia, for whom individualized treatment strategies may be warranted.

The term sarcopenia, derived from the Greek ‘sarx’ (flesh) and ‘penia’ (loss), was proposed by Rosenberg in 1997 to define age-associated loss of skeletal muscle mass and strength.^7^ Currently, it is widely recognized as a progressive skeletal muscle disorder, with incidence and diagnostic criteria that vary across populations. Sarcopenia is closely linked to frailty, which represents a multidimensional syndrome of reduced physiological reserve associated with adverse cardiovascular outcomes.^8^ Although sarcopenia and frailty often overlap and are sometimes regarded as interchangeable, sarcopenia represents a distinct clinical entity that can develop even in relatively younger individuals and has been recognized as an independent risk factor for adverse outcomes across various clinical settings.^9–12^ In Europe, sarcopenia is diagnosed according to the European Working Group on Sarcopenia in Older People (EWGSOP2) criteria, which consider not only low skeletal muscle mass but also reduced muscle strength and function.^13^ Sarcopenia has been associated with increased risks of fractures, osteoporosis, hospitalization, poor quality of life, and all-cause mortality. Moreover, its potential relationship with cardiovascular disease has been widely explored.^14,15^

Recent evidence from a UK Biobank study involving a predominantly Caucasian cohort found that sarcopenia was independently associated with an increased long-term risk of incident AF. Notably, this association appeared stronger among younger individuals, females, and those with valvular heart disease.^16^

However, evidence on how sarcopenia affects clinical outcomes, especially bleeding risk among patients with AF subjected to anticoagulation therapy, remains scarce, highlighting a critical gap in current research. Therefore, the present study aimed to examine the association between sarcopenia and key clinical outcomes, including all-cause mortality, thromboembolic events, and bleeding, in patients with AF using data from the UK Biobank, with the ultimate goal of informing risk stratification and guiding personalized management.

## Methods

### Data source and study population

**Figure 1:**
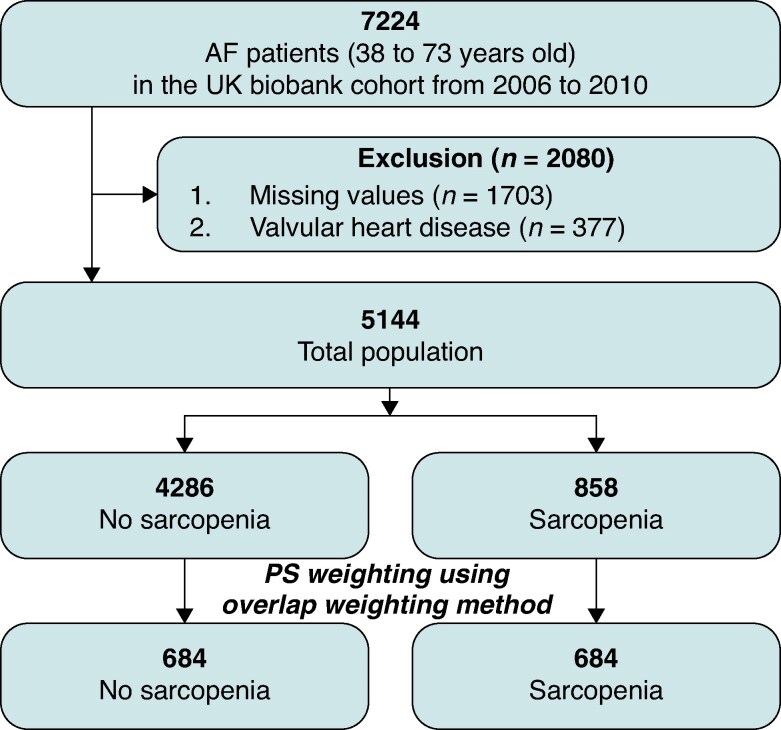
Flow chart of participant selection and analysis in the UK Biobank cohort.

This study utilized data from the UK Biobank, a prospective, large-scale cohort that recruited over 500 000 individuals aged 40–69 years during the 2006–10 enrolment period to examine the effects of genetic, socioeconomic, lifestyle, and environmental factors on various diseases and long-term health outcomes. Recruitment was conducted across 22 centres in the UK, with ∼5.5% of 9.2 million invited individuals participating in the initial assessment.^17^ The participants underwent comprehensive baseline assessments, including questionnaires, physical examinations, biochemical tests, imaging, and genotyping. Longitudinal health outcomes were tracked through linkage with the National Electronic Health Record database. The methodology of the UK Biobank has been described in previous studies.^18,19^ Among a total of 502 421 participants enrolled in the UK Biobank, 7224 individuals with a diagnosis of AF at baseline were identified and included in this study. Participants with missing values in baseline covariates used for propensity score modeling (*n* = 1703) were excluded. In addition, those with valvular heart disease, including mitral valve stenosis or prosthetic valve status (*n* = 377; International Classification of Disease (ICD)-10 codes: I050, I052, I342), were excluded. The final analytical cohort comprised 5144 participants (*Figure 1*). Frailty was assessed using the hospital frailty risk score, which was calculated based on diagnostic codes as previously described.^20^ Participants were categorized into three frailty risk groups according to the established thresholds: low risk (<5 points), intermediate risk (5–15 points), and high risk (>15 points). Socioeconomic status was evaluated using the Townsend deprivation index, which reflects material deprivation based on employment, housing, and car ownership, and was categorized into quartiles (25th, 50th, and 75th percentiles).^21^

### Definition of sarcopenia

Currently, sarcopenia can be defined using two major diagnostic frameworks: the EWGSOP2 and the Asian Working Group for Sarcopenia (2019).^13,22^ In this study, sarcopenia was determined according to the EWGSOP2 diagnostic standards. In line with this framework, participants with low muscle strength and/or low muscle quantity were classified as having sarcopenia, thereby encompassing both possible and confirmed stages. Muscle strength was assessed by the handgrip test, and muscle quantity was estimated using bioelectrical impedance analysis (BIA) data from the UK Biobank. Appendicular lean soft tissue (ALST) was derived from appendicular fat-free mass (AFFM) calculated using the following formula: ALST = (0.958 × AFFM) − (0.166 × *S*) − 0.308, where *S* represents sex (0 for female and 1 for male). The skeletal muscle mass index (SMI) was obtained by dividing ALST by height squared (kg/m²). Low muscle quantity was defined as SMI <6.95 kg/m² for males and <5.30 kg/m² for females.

### Ethical approval

The study protocol was approved by the Institutional Review Board of Yeungnam University Medical Center (IRB No. 2025-01-001). All procedures adhered to the ethical principles outlined in the 1964 Declaration of Helsinki and its subsequent revisions. As the study was a retrospective analysis of anonymized UK Biobank data, the requirement for informed consent was waived.

### Ascertainment of clinical outcomes

The primary outcome comprised all-cause mortality, stroke or systemic embolism, major bleeding or clinically relevant non-major bleeding (as defined by the International Society on Thrombosis and Haemostasis),^23^ and hospitalization due to heart failure. Each component was also assessed individually as a secondary outcome. Clinical outcome data were obtained using inpatient and outpatient records containing relevant ICD-10 codes, supplemented by self-reported non-cancer illness codes in the UK Biobank database in accordance with validated methods used in prior analyses, which rely on multiple records to improve the accuracy of cardiovascular disease identification.^24–27^ Detailed definitions of comorbidities and outcomes are presented in Supplementary material online, *Tables S1* and *S2*, respectively.

### Statistical methods

Group comparisons for categorical variables were carried out using either Pearson’s *χ*^2^ test or Fisher’s exact test, depending on the expected frequencies. For continuous variables, we applied either the independent-samples *t*-test or the Mann–Whitney *U* test according to data normality. Adjustment for baseline differences between groups was performed by propensity score (PS) weighting using the overlap weighting method. The PS model included the following covariates: age, sex, body mass index (BMI), systolic and diastolic blood pressure, CHA_2_DS_2_-VASc score, hypertension, diabetes mellitus, heart failure, ischaemic stroke or transient ischaemic attack (TIA), prior myocardial infarction, hyperthyroidism, hypothyroidism, osteoporosis, dyslipidaemia, end-stage renal disease (ESRD), chronic obstructive pulmonary disease (COPD), and a history of malignant neoplasm. Outcomes were compared between groups by Kaplan–Meier analysis. Furthermore, Cox proportional hazard regression analysis was conducted to assess the risk of primary and secondary outcomes, and the results are expressed as hazard ratios (HRs) with 95% confidence intervals (CIs). Variables with *P* < 0.10 in univariable analysis were included in the multivariable model. Proportional hazard assumptions were verified using scaled Schoenfeld residuals. Statistical significance was defined as a two-tailed *P* < 0.05. Analyses were performed using R (version 4.3.2; R Foundation for Statistical Computing, Vienna, Austria).

## Results

### Baseline characteristics

The baseline characteristics of the study population, stratified by sarcopenia status, are presented in *Table 1*. Prior to PS weighting, the analysis included 5144 individuals diagnosed with AF, of whom 858 patients (16.7%) had sarcopenia. In comparison with individuals without sarcopenia, those with sarcopenia tended to be older (65.0 vs. 64.0) and comprised a higher proportion of females (29.3% vs. 23.1%). Additionally, the sarcopenia group had significantly higher CHA_2_DS_2_-VASc scores and a greater prevalence of comorbidities, including heart failure, hypertension, diabetes mellitus, TIA or stroke, hypothyroidism, osteoporosis, dyslipidaemia, ESRD, and COPD (*Table 1*). Following PS weighting with the overlap weighting approach, baseline characteristics included in the PS model appeared comparable between the sarcopenia and non-sarcopenia groups [all standardized mean differences (SMD) < 0.001; *Table 1* and Supplementary material online, *Figure S1*].

**Table 1:** Baseline characteristics of patients with AF stratified by sarcopenia status

	Overall population			Propensity score-weighted population			
	No sarcopenia (*n* = 4286)	Sarcopenia (*n* = 858)	*P*-value	No sarcopenia (*n* = 684)	Sarcopenia (*n* = 684)	*P*-value	SMD
Age, years	64.0 (60.0, 67.0)	65.0 (62.0, 68.0)	<0.001	65.0 (61.0, 68.0)	65.0 (62.0, 68.0)	0.373	<0.001
Male	3297 (76.9)	607 (70.7)	<0.001	490.1 (71.7)	490.1 (71.7)	1.000	<0.001
BMI, kg/m^2^	28.37 (25.8, 31.8)	28.24 (24.2, 32.5)	0.006	27.97 (25.3, 31.4)	28.44 (24.6, 32.6)	0.892	<0.001
SBP, mmHg	137.5 (125.0, 150.0)	135.0 (123.0, 150.5)	0.027	136.5 (124.0, 149.0)	135.5 (123.5, 151.0)	0.716	<0.001
DBP, mmHg	82.0 (75.0, 89.5)	80.0 (73.0, 88.0)	<0.001	81.0 (73.5, 88.0)	80.0 (73.5, 88.5)	0.803	<0.001
Charlson comorbidity index	1.0 (0.0, 2.0)	1.0 (0.0, 2.0)	<0.001	1.0 (0.0, 2.0)	1.0 (0.0, 2.0)	0.015	0.098
Hospital frailty risk categories			<0.001			0.382	0.053
Low risk	3842 (89.6)	703 (81.9)		570.7 (83.5)	574.5 (84.0)		
Intermediate risk	417 (9.7)	134 (15.6)		104.0 (15.2)	96.3 (14.1)		
High risk	27 (0.6)	21 (2.4)		9.2 (1.3)	13.0 (1.9)		
Townsend index quartiles, *n* (%)			<0.001			0.469	0.061
Q1 (less deprived)	1096 (25.6)	189 (22.0)		148.8 (21.8)	156.5 (22.9)		
Q2	1072 (25.0)	198 (23.1)		165.2 (24.2)	161.5 (23.6)		
Q3	1091 (25.5)	208 (24.2)		177.8 (26.0)	162.4 (23.7)		
Q4 (most deprived)	1027 (24.0)	263 (30.7)		192.0 (28.1)	203.5 (29.8)		
Current smoker	301 (7.0)	76 (8.9)	0.070	50.6 (7.4)	58.0 (8.5)	0.289	0.040
CHA_2_DS_2_-VASc score	2.0 (1.0, 2.0)	2.0 (1.0, 3.0)	<0.001	2.0 (1.0, 3.0)	2.0 (1.0, 3.0)	0.879	<0.001
Heart failure	541 (12.6)	134 (15.6)	0.021	102.2 (14.9)	102.2 (14.9)	1.000	<0.001
Hypertension	2722 (63.5)	606 (70.6)	<0.001	475.8 (69.6)	475.8 (69.6)	1.000	<0.001
Diabetes mellitus	537 (12.5)	156 (18.2)	<0.001	116.7 (17.1)	116.7 (17.1)	1.000	<0.001
Ischaemic stroke or TIA	223 (5.2)	69 (8.0)	0.001	50.0 (7.3)	50.0 (7.3)	1.000	<0.001
Previous MI	417 (9.7)	102 (11.9)	0.064	78.7 (11.5)	78.7 (11.5)	1.000	<0.001
Hyperthyroidism	99 (2.3)	25 (2.9)	0.352	18.9 (2.8)	18.9 (2.8)	1.000	<0.001
Hypothyroidism	256 (6.0)	69 (8.0)	0.028	52.2 (7.6)	52.2 (7.6)	1.000	<0.001
Osteoporosis	67 (1.6)	27 (3.1)	0.003	17.2 (2.5)	17.2 (2.5)	1.000	<0.001
Dyslipidaemia	1568 (36.6)	375 (43.7)	<0.001	290.3 (42.5)	290.3 (42.5)	1.000	<0.001
ESRD or CKD	221 (5.2)	66 (7.7)	0.004	47.4 (6.9)	47.4 (6.9)	1.000	<0.001
COPD	161 (3.8)	69 (8.0)	<0.001	46.6 (6.8)	46.6 (6.8)	1.000	<0.001
History of malignant neoplasm	445 (10.4)	105 (12.2)	0.122	80.6 (11.8)	80.6 (11.8)	1.000	<0.001
Antiplatelet agent use	2028 (47.3)	408 (47.6)	0.929	324.8 (47.5)	323.2 (47.3)	0.902	0.005
Aspirin use	1954 (45.6)	382 (44.5)	0.592	311.2 (45.5)	302.2 (44.2)	0.488	0.027
Vitamin K antagonist use	1500 (35.0)	338 (39.4)	0.016	257.8 (37.7)	268.2 (39.2)	0.413	0.031
No antithrombotic therapy	868 (20.3)	153 (17.8)	0.115	121.5 (17.8)	123.9 (18.1)	0.814	0.009

Values are presented as medians [Q1 and Q3 quartiles (25th and 75th percentiles)] or numbers (%).

Socioeconomic status was categorized according to the Townsend deprivation index into quartiles: Q1 (least deprived), Q2, Q3, and Q4 (most deprived).

CKD, chronic kidney disease; COPD, chronic obstructive pulmonary disease; DBP, diastolic blood pressure; ESRD, end-stage renal disease; MI, myocardial infarction; SBP, systolic blood pressure; SMD, standardized mean difference; TIA, transient ischaemic attack

### Primary and secondary outcomes

**Figure 2:**
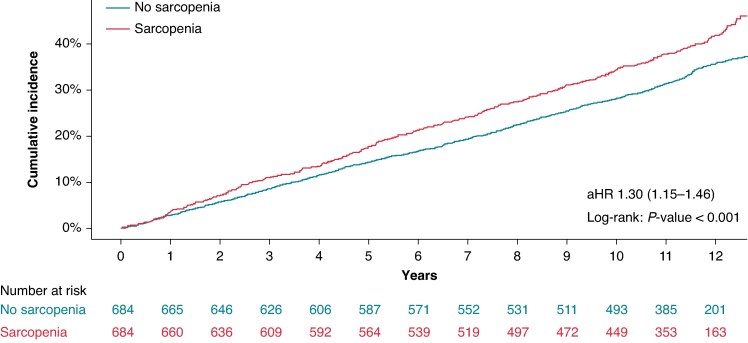
Kaplan–Meier analysis of the primary composite outcome stratified by sarcopenia status in patients with AF.

**Figure 3:**
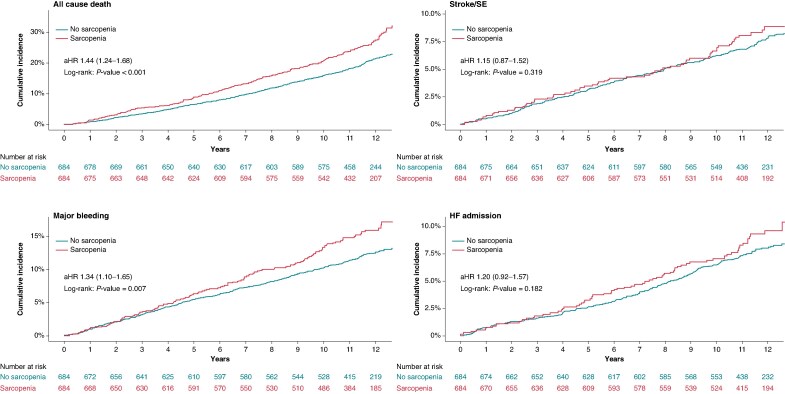
Kaplan–Meier analysis of secondary outcomes stratified by sarcopenia status in patients with AF.

The incidence rate of the primary composite outcome was higher in the sarcopenia group than in the non-sarcopenia group (43.9 vs. 35.1 events per 1000 person-years), and this difference was statistically significant (*P* = 0.014). Kaplan–Meier survival curves similarly demonstrated a significant divergence between the two groups, with the sarcopenia group exhibiting a significantly greater cumulative incidence of the primary outcome (log-rank *P* < 0.001). In Cox proportional hazard regression analysis, sarcopenia remained independently associated with an elevated risk (30%) of the primary outcome (adjusted HR, 1.30; 95% CI 1.15–1.46; *Table 2* and *Figure 2*). Among individual components of the composite outcome, the risk of all-cause mortality was significantly higher in the sarcopenia group (aHR, 1.44; 95% CI 1.24–1.68; log-rank *P* < 0.001). Bleeding events were also significantly more frequent in patients with sarcopenia (aHR, 1.34; 95% CI 1.10–1.65; log-rank *P* = 0.007). Although stroke/systemic embolism (aHR, 1.15; 95% CI 0.87–1.52; log-rank *P* = 0.319) and heart failure admission (aHR, 1.20; 95% CI 0.92–1.57; log-rank *P* = 0.182) were more common in patients with sarcopenia, the results were not statistically significant (*Table 2*, *Figure 3*).

**Table 2:** Incidence and risk of the primary and secondary outcomes of the overall and propensity score-weighted population

	Overall population			Propensity score-weighted population		
	Cases, *n* (%)	Incidence (/1000 PYRs)	Adjusted HR (95% CI)	Cases, *n* (%)	Incidence (/1000 PYRs)	Adjusted HR (95% CI)
Primary outcome						
No sarcopenia	1294 (30.2%)	29.6	Reference	237 (34.7%)	35.1	Reference
Sarcopenia	367 (42.8%)	46.0	1.28 (1.14–1.45)	282 (41.3%)	43.9	1.30 (1.15–1.46)
Secondary outcomes						
*All-cause death*						
No sarcopenia	738 (17.2%)	15.6	Reference	142 (20.8%)	19.2	Reference
Sarcopenia	249 (29.0%)	28.0	1.43 (1.24–1.66)	189 (27.6%)	26.4	1.44 (1.24–1.68)
*Major bleeding events*						
No sarcopenia	444 (10.4%)	9.8	Reference	78 (11.3%)	11.0	Reference
Sarcopenia	126 (14.7%)	15.1	1.32 (1.08–1.61)	97 (14.2%)	14.4	1.34 (1.10–1.65)
*Stroke or systemic embolic events*						
No sarcopenia	258 (6.0%)	5.6	Reference	47 (6.9%)	6.6	Reference
Sarcopenia	67 (7.8%)52 (7.6%)	7.8	1.13 (0.86–1.48)	52 (7.6%)	7.5	1.15 (0.87–1.52)
*Heart failure admission*						
No sarcopenia	246 (5.7%)	5.3	Reference	50 (7.2%)	6.8	Reference
Sarcopenia	73 (8.5%)	8.4	1.19 (0.91–1.55)	56 (8.2%)	8.0	1.20 (0.92–1.57)

CI, confidence interval; PYRs, person-years

### Sensitivity analyses

**Figure 4:**
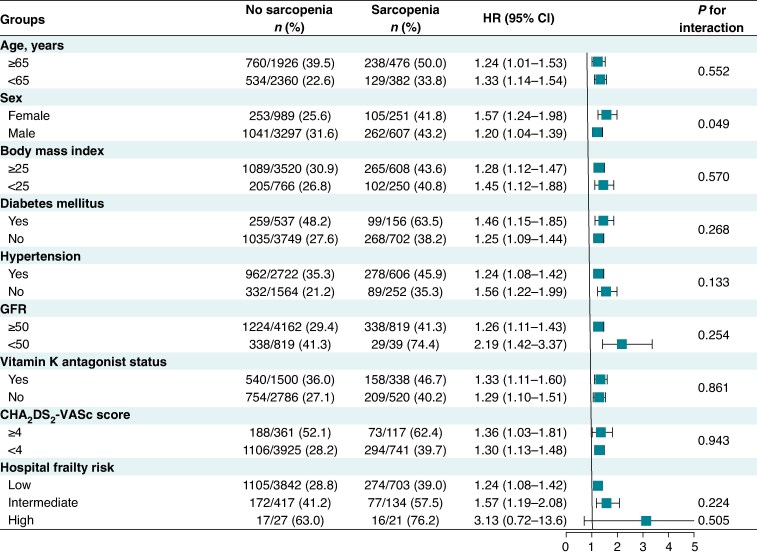
Subgroup analysis for the primary composite outcome according to sarcopenia status.

Sensitivity analyses were conducted to evaluate the robustness of the association between sarcopenia and the primary outcome. Analyses using muscle quantity (measured by BIA) and muscle quality (handgrip strength) were performed separately to assess their individual associations with the primary outcome. Despite the different definitions, both analyses consistently demonstrated that sarcopenia was associated with adverse outcomes. Specifically, muscle quality-based sarcopenia was significantly associated with the primary outcome both before and after adjustment (unadjusted HR 1.58, 95% CI 1.40–1.79; adjusted HR 1.26, 95% CI 1.11–1.42), and muscle quantity-based sarcopenia was likewise significantly associated with the primary outcome (unadjusted HR 1.42, 95% CI 1.08–1.87; adjusted HR 1.53, 95% CI 1.14–2.04) (see Supplementary material online, *Table S3*). Additionally, subgroup analyses were performed across clinically relevant categories, including age, sex, BMI, diabetes mellitus, hypertension, reduced estimated glomerular filtration rate (eGFR; <50 mL/min/1.73 m²), current use of vitamin K antagonists, CHA₂DS₂-VASc score >4, and hospital frailty risk. Across all subgroups, sarcopenia remained consistently associated with an increased risk of the primary outcome (*Figure 4*), reinforcing the robustness of its prognostic impact. A numerically stronger association was observed among females, and the association remained generally consistent across other subgroups, including those with impaired renal function, without hypertension, and across frailty categories. A borderline interaction with sex (*P* = 0.049) suggested that the effect may be more pronounced in females. Furthermore, a sensitivity analysis for the bleeding outcome was performed across relevant clinical subgroups. Sarcopenia consistently demonstrated an association with poorer outcomes, including among patients stratified by vitamin K antagonist use (see Supplementary material online, *Figure S2*).

## Discussion

In this population-based cohort study, we identified several key findings: (i) even after PS weighting, sarcopenia was independently associated with an increased risk of the primary composite outcome; (ii) in secondary analyses, sarcopenia was significantly associated with increased risks of all-cause mortality and major bleeding; and (iii) these associations remained consistent in multiple prespecified subgroups.

### Effect of sarcopenia on cardiovascular disease

Although sarcopenia was historically viewed as a musculoskeletal condition, it is now recognized as a systemic disorder that shares several pathophysiological mechanisms with cardiovascular disease, including anabolic resistance, mitochondrial dysfunction, and chronic inflammation.^28,29^ Sarcopenia has been independently linked to adverse cardiovascular outcomes such as acute decompensated heart failure, congestive heart failure, and coronary atherosclerosis.^30–32^ The biological continuum between sarcopenia and cardiovascular disease may be driven by insulin resistance, oxidative stress, and neurohormonal activation, which together contribute to metabolic and vascular dysfunction.^29,33–35^ Reductions in skeletal muscle mass and function may lead to arterial stiffness, hypertension, and ischaemic heart disease.^34,35^ These mechanisms support the emerging view of sarcopenia as a modifiable contributor to cardiovascular risk. Although accumulating data indicate a potential link between sarcopenia and incident AF,^16,36^ few studies have investigated the effect of sarcopenia on the clinical outcomes of patients with AF. Our study addresses this gap by demonstrating that sarcopenia represents an independent determinant of adverse outcomes, including mortality and bleeding, in AF populations.

### Anticoagulation and bleeding risk in patients with sarcopenia and AF

In patients with AF, anticoagulation therapy remains the cornerstone of stroke prevention. Although clinical guidelines advise that bleeding risk should not be the sole factor in anticoagulation decisions, recently published European guidelines emphasize the importance of actively managing modifiable bleeding risk factors, elevating this to a Class I recommendation. A multidisciplinary approach is encouraged to optimize care for patients with potentially modifiable bleeding risk factors.^3,4^ Established bleeding risk scores commonly include age and prior bleeding as key predictors.^37,38^ Increasing attention has been paid to frailty as an emerging factor influencing bleeding and anticoagulation decisions, particularly in optimizing safety and treatment selection in older or high-risk patients.^39–43^ Likewise, low body weight has been associated with increased bleeding risk, prompting dosing adjustments for non-vitamin K antagonist oral anticoagulants (NOACs).^3,4,44,45^ Although no definitive pathophysiological mechanism directly linking sarcopenia to bleeding tendency has been established, sarcopenia may serve as a surrogate marker for frailty, malnutrition, and decreased physiological reserve, all of which are relevant contributors to bleeding complications. Despite its relevance, sarcopenia remains an underrecognized factor in the context of AF management. Sarcopenia is often considered overlapping with, and sometimes interchangeable with, frailty. However, our study demonstrated that sarcopenia represents a distinct clinical entity independent of frailty. Even after propensity score weighting achieved baseline balance between groups, sarcopenia remained significantly associated with a higher risk of adverse primary outcomes, and no significant interaction was observed across frailty categories in the subgroup analysis. Moreover, sarcopenia was also associated with worse bleeding outcomes. Collectively, these findings indicate that sarcopenia should be regarded as an independent risk factor, distinct from frailty, in patients with AF. Both the ABC pathway and the recently proposed AF-CARE framework emphasize multidisciplinary, integrated management and individualized care for this population.^3–5^ In particular, these approaches highlight the importance of identifying and managing risk factors that may increase bleeding risk, a key component of stroke prevention strategies. Similar to how dose adjustments of DOACs and tailored therapy are recommended for frail individuals, sarcopenia should likewise be recognized as a potential risk factor that warrants clinical attention and individualized management within these frameworks. These results support the incorporation of sarcopenia screening and interdisciplinary care into routine AF management, particularly for patients receiving anticoagulation therapy. Further prospective studies are needed to determine whether targeted interventions for sarcopenia can improve outcomes in this high-risk population.

## Limitations

This study has several limitations. First, as a retrospective observational study, it is susceptible to unmeasured confounding. Second, the use of administrative data (UK Biobank) may introduce diagnostic misclassification. Nevertheless, we addressed this limitation by relying on validated definitions from previous studies.^1,46–49^ Third, despite using a large national dataset, the number of participants in our study was limited by the relatively low incidence rates of AF and sarcopenia. Nevertheless, our study is among the first to investigate the association between sarcopenia and adverse clinical outcomes in patients with AF utilizing UK Biobank data, which is a novelty of our study. Fourth, the generalizability of our findings may be limited due to the demographic characteristics of the UK Biobank cohort, which predominantly comprises White individuals who are generally healthier and more health-conscious than the general population, reflecting a potential ‘healthy volunteer’ bias. Lastly, the enrolment period of the UK Biobank cohort (2006–10) preceded the introduction of direct oral anticoagulants (DOACs), when anticoagulation therapy was primarily limited to vitamin K antagonists.^50^ Although this temporal context may limit the direct applicability of our findings to the current DOAC era, sarcopenia remained consistently associated with adverse outcomes regardless of VKA use. This finding reinforces the prognostic importance of sarcopenia and underscores the need to recognize it as a clinically relevant factor in the management of patients with AF.^17^

## Conclusions

In this large population-based cohort study, sarcopenia was independently associated with an increased risk of adverse clinical outcomes, including all-cause mortality and major bleeding, in patients with AF. These findings suggest that sarcopenia may be a clinically relevant and potentially modifiable risk factor in this population. Therefore, incorporating routine sarcopenia screening into AF management may enhance risk stratification and support personalized treatment approaches, especially for patients receiving anticoagulation therapy.

## Supplementary Material

euaf286_Supplementary_Data

## Data Availability

This study used data from the UK Biobank. The authors are not permitted to share the data directly. Access to the UK Biobank dataset can be obtained by applying via the UK Biobank (https://www.ukbiobank.ac.uk). Additional analytic code and documentation can be provided by the corresponding author upon reasonable request. Data sharing is subject to the policies and ethical guidelines of UK Biobank and may be restricted accordingly.
