# Supplementary material for: Sarcopenia in atrial fibrillation: a risk factor for adverse outcomes in a UK Biobank study
Source: Europace. 2025 Nov 8;27(12):euaf286. doi: 10.1093/europace/euaf286 (PMC12722030; doi:10.1093/europace/euaf286)

**Supplementary Materials**

**Supplementary Methods**

**Supplementary table 1.** Definitions of comorbidities

**Supplementary table 2.** Definitions used to define the outcomes

**Supplementary table 3.** Sensitivity analyses for the association between sarcopenia and the primary composite outcome

**Supplementary figure 1.** Propensity Score Distribution Before and After Overlap Weighting in Sarcopenia and Control Groups

**Supplementary figure 2.** Forest Plot of Subgroup Analysis for Bleeding Comparing No Sarcopenia and Sarcopenia in Patients with Atrial Fibrillation

**Supplementary table 1.** Definitions of comorbidities

| **Comorbidities** | **UK Biobank** | |
| --- | --- | --- |
|  | **Definitions** | **Used codes or conditions** |
| Atrial fibrillation | Defined from U.K. Biobank self-report or diagnosis^a^ | Self-reported non-cancer illness code: 1471, 1483  ICD-10: I48 |
| Hypertrophic cardiomyopathy | Defined from U.K. Biobank self-report or diagnosis | Self-reported non-cancer illness code: 1588  ICD-10: I42.1, I42.2 |
| Heart failure | Defined from U.K. Biobank self-report or diagnosis^a^ | Self-reported non-cancer illness code: 1076  ICD-10: I11.0, I50, I97.1 |
| Ischemic stroke | Defined from U.K. Biobank self-report or diagnosis^a^ | Self-reported non-cancer illness code: 1583  ICD-10: I63, I64 |
| TIA | Defined from U.K. Biobank self-report or diagnosis^a^ | Self-reported non-cancer illness code: 1082  ICD-10: G45 |
| Hemorrhagic stroke | Defined from U.K. Biobank self-report or diagnosis^a^ | Self-reported non-cancer illness code: 1086, 1491  ICD-10: I60, I61, I62 |
| Diabetes mellitus | Defined from U.K. Biobank self-report or diagnosis^a^ | Self-reported non-cancer illness code: 1220, 1222, 1223, 1521  ICD-10: E10, E11, E12, E13, E14 |
| Hypertension | Defined from U.K. Biobank self-report or diagnosis^a^ | Self-reported non-cancer illness code: 1065, 1072  ICD-10: I10, I11, I12, I13, I15 |
| Previous myocardial infarction (MI) | Defined from U.K. Biobank self-report or diagnosis^a^ | Self-reported non-cancer illness code: 1075  ICD-10: I21, I22, I25.2 |
| Peripheral arterial disease | Defined from U.K. Biobank self-report or diagnosis^a^ | Self-reported non-cancer illness code: 1067, 1087  ICD-10: I70, I71 |
| Dyslipidemia | Defined from U.K. Biobank self-report or diagnosis^a^ | Self-reported non-cancer illness code: 1473  ICD-10: E78 |
| COPD | Defined from U.K. Biobank self-report or diagnosis^a^ | Self-reported non-cancer illness code: 1112, 1113, 1472  ICD-10: J42, J43(except J43.0), J44 |
| Chronic renal failure | Defined from eGFR (if laboratory value was not available, self-report or diagnosis code was used) | eGFR <60 mL/min per 1.73 m^2^  Self-reported non-cancer illness code:1192, 1194  ICD-10: N18, N19 |
| ESRD | Defined from U.K. Biobank self-report or procedure codes related to renal replacement therapy (hemodialysis, peritoneal dialysis, or kidney transplant) | Self-reported non-cancer illness code: 1193, 1195, 1580, 1581, 1582  Procedure codes: L74, M01, M02.3, M08.4, M17, X40, X41, X42 |
| Malignancy | Defined from U.K. Biobank self-report or diagnoses of cancer (non-benign) | Self-reported cancer illness code: all  ICD-10: C00-C97 |
| Potential absence of non-valvular atrial fibrillation | Defined from U.K. Biobank self-report / any diagnoses of mitral stenosis or procedure codes for heart valve surgery | Self-reported non-cancer illness code: 1489  ICD-10: I05.0, I05.2, I34.2, Z95.2-4  Procedure codes: K25, K26, K27, K28, K29, K30, K31, K32, K33, K34, K35 |
| Hyperthyroidism | Defined from U.K. Biobank self-report or diagnosis^a^ | Self-reported non-cancer illness code: 1225, 1522  ICD-10: E05 |
| Hypothyroidism | Defined from U.K. Biobank self-report or diagnosis^a^ | Self-reported non-cancer illness code: 1226  ICD-10: E03 |
| Venous thrombosis including pulmonary embolism | Defined from U.K. Biobank self-report or diagnosis^a^ | Self-reported non-cancer illness code: 1068, 1093, 1094  ICD-10: I26, I80.1, I80.2, I80.3, I80.8, I80.9, I81, I82, I63.6, O22.3, O22.5, O87.1, O87.3, G80 |

^a^To ensure accuracy, comorbidities were established based on more than one hospital inpatient or two outpatient records of ICD-10 codes in the database (primary care in the United Kingdom).

**Supplementary table 2.** Definitions used to define the outcomes

| **Comorbidities** | **UK Biobank** | |
| --- | --- | --- |
|  | **Definitions** | **Used codes or conditions** |
| New-onset atrial fibrillation (AF) | Defined from diagnosis^a^ or related death without previous history of AF | ICD-10: I48 |
| Heart failure admission | Defined from hospital-inpatient diagnosis (primary diagnosis), or related death | ICD-10: I11.0, I50, I97.1 |
| Stroke admission | Defined from hospital-inpatient diagnosis (primary diagnosis), or related death | ICD-10: I63, I64  Procedure codes for brain imaging: U051, U052, U053, U114 |
| All-cause death | Data related to death were confirmed by the death registries of United Kingdom | The cause of death was determined based on the ICD-10 code written on the death certificate. |

^a^To ensure accuracy, comorbidities were established based on more than one hospital inpatient or two outpatient records of ICD-10 codes in the database (primary care in the United Kingdom).

**Supplementary table 3.** Sensitivity analyses for the association between sarcopenia and the primary composite outcome

| **Definition of sarcopenia** | **Model** | **HR (95% CI)** | **P value** |
| --- | --- | --- | --- |
| **Muscle quality**  (Handgrip strength) | Unadjusted | 1.58 (1.40–1.79) | <0.001 |
|  | Adjusted | 1.26 (1.11–1.42) | <0.001 |
| **Muscle quantity**  (Measured by BIA) | Unadjusted | 1.42 (1.08–1.87) | 0.013 |
|  | Adjusted | 1.53 (1.14–2.04) | <0.001 |

BIA, bioelectrical impedance analysis; CI, confidence interval; HR, hazard ratio.

The adjusted model includes age, sex, BMI, smoking, blood pressure, comorbidities, renal function (eGFR), socioeconomic status, and hospital frailty risk.

**Supplementary figure 1.** Propensity Score Distribution Before and After Overlap Weighting in Sarcopenia and Control Groups


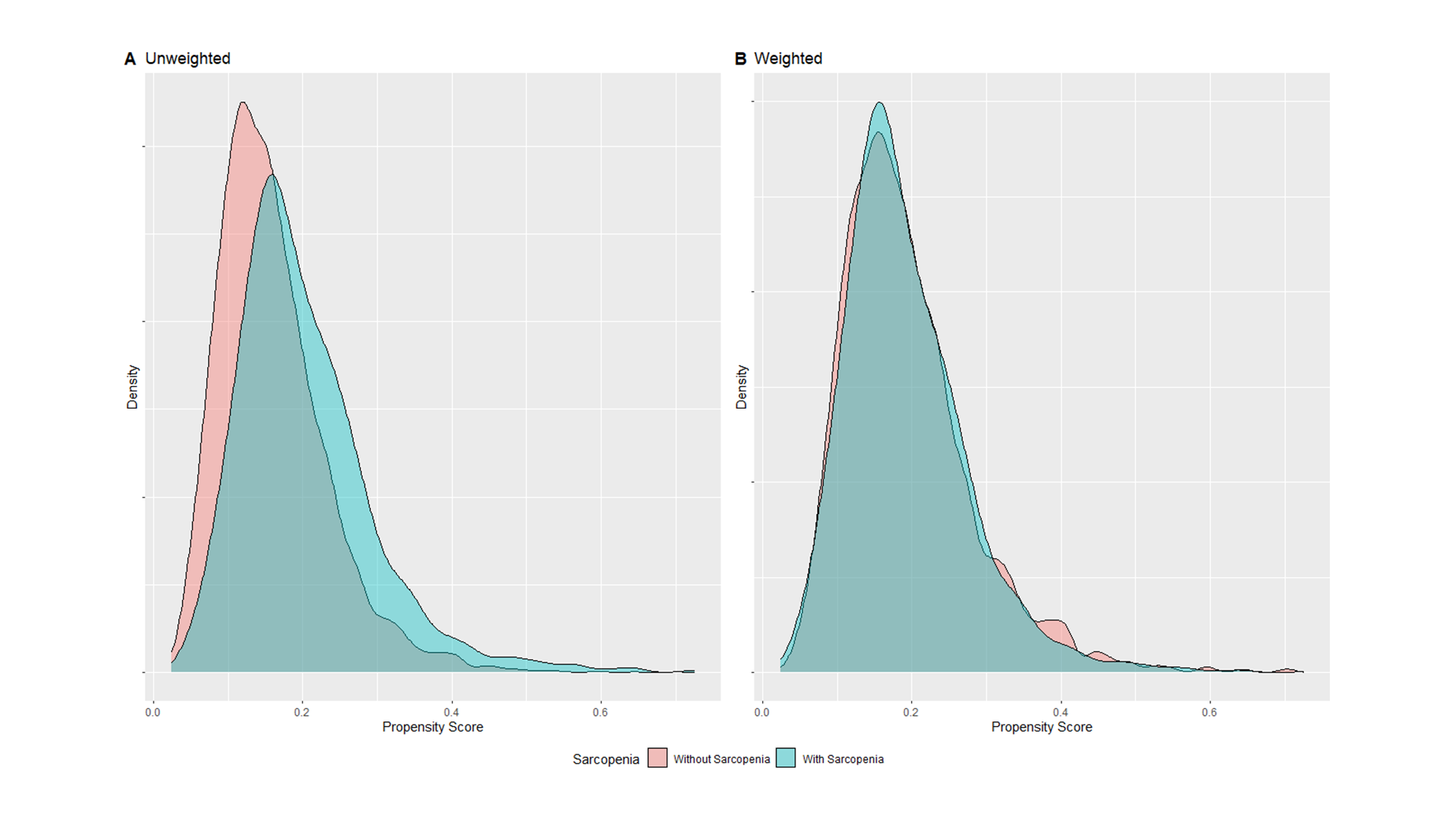


**Supplementary figure 2.** Forest Plot of Subgroup Analysis for Bleeding Comparing No Sarcopenia and Sarcopenia in Patients with Atrial Fibrillation


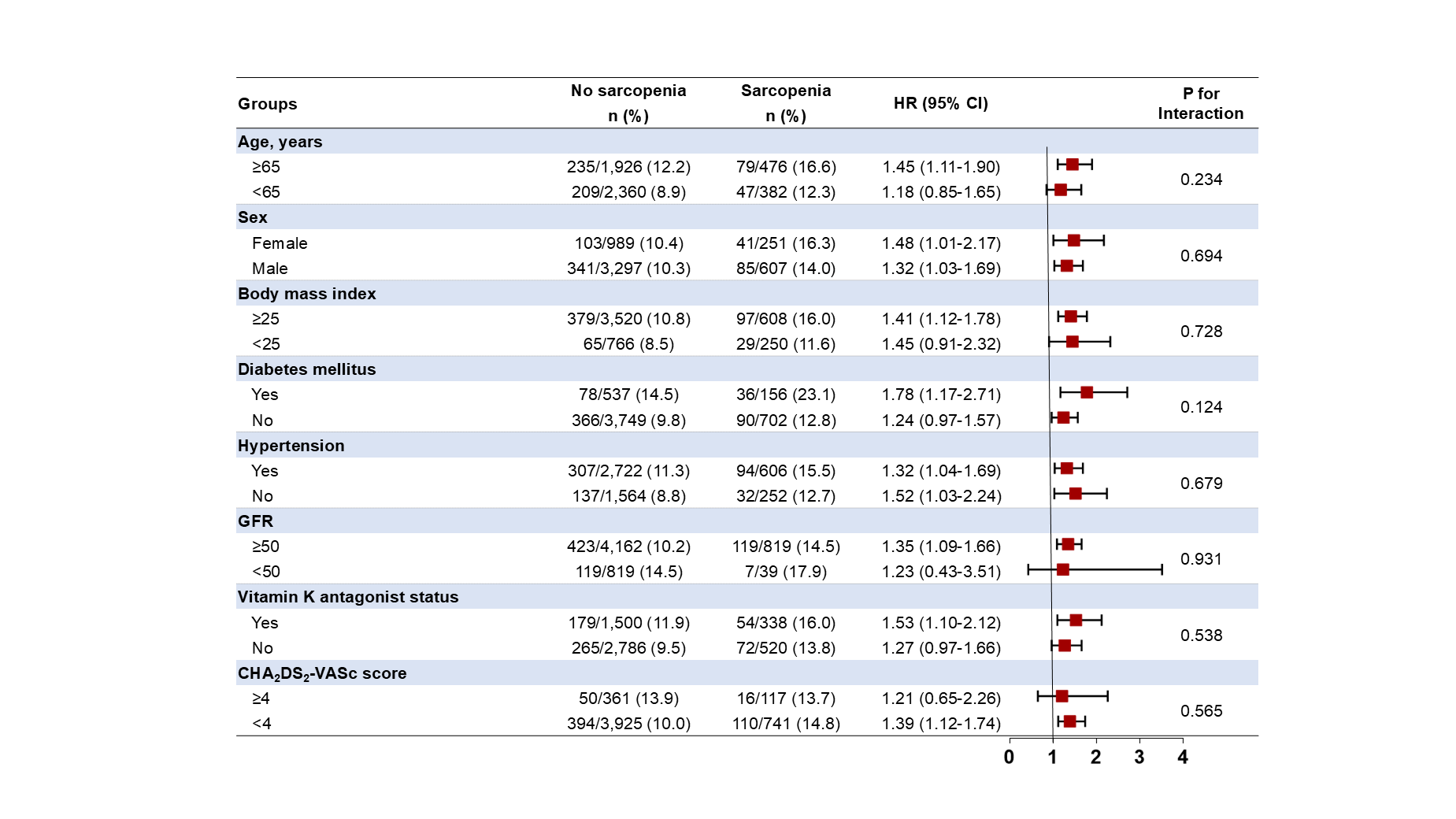

Supplement: euaf286_Supplementary_Data [file euaf286_supplementary_data.docx]
